# STI-GMaS: an open-source environment for simulation of sexually-transmitted infections

**DOI:** 10.1186/1752-0509-8-66

**Published:** 2014-06-12

**Authors:** Martin R Nelson, Kelly J Sutton, Bindi S Brook, Dann G Mallet, Daniel P Simpson, Roger G Rank

**Affiliations:** 1 School of Science and Technology, Nottingham Trent University, Clifton Campus, Nottingham NG11 8NS, UK; 2 School of Mathematical Sciences, University of Nottingham, Nottingham NG7 2RD, UK; 3 Department of Infectious Disease Epidemiology, Imperial College London, St. Mary’s Campus, London W2 1PG, UK; 4 Mathematical Sciences School, Science and Engineering Faculty, Queensland University of Technology, Brisbane, Australia; 5 Department of Mathematical Sciences, Norwegian University of Science and Technology, Trondheim, Norway; 6 Department of Microbiology and Immunology, University of Arkansas for Medical Sciences and Arkansas Children’s Hospital Research Institute, Little Rock, Arkansas, USA

**Keywords:** Sexually-transmitted infections, Cellular automata, Hybrid models, Computational software

## Abstract

**Background:**

Sexually-transmitted pathogens often have severe reproductive health implications if treatment is delayed or absent, especially in females. The complex processes of disease progression, namely replication and ascension of the infection through the genital tract, span both extracellular and intracellular physiological scales, and in females can vary over the distinct phases of the menstrual cycle. The complexity of these processes, coupled with the common impossibility of obtaining comprehensive and sequential clinical data from individual human patients, makes mathematical and computational modelling valuable tools in developing our understanding of the infection, with a view to identifying new interventions. While many within-host models of sexually-transmitted infections (STIs) are available in existing literature, these models are difficult to deploy in clinical/experimental settings since simulations often require complex computational approaches.

**Results:**

We present STI-GMaS (Sexually-Transmitted Infections – Graphical Modelling and Simulation), an environment for simulation of STI models, with a view to stimulating the uptake of these models within the laboratory or clinic. The software currently focuses upon the representative case-study of *Chlamydia trachomatis*, the most common sexually-transmitted bacterial pathogen of humans. Here, we demonstrate the use of a hybrid PDE–cellular automata model for simulation of a hypothetical *Chlamydia* vaccination, demonstrating the effect of a vaccine-induced antibody in preventing the infection from ascending to above the cervix. This example illustrates the ease with which existing models can be adapted to describe new studies, and its careful parameterisation within STI-GMaS facilitates future tuning to experimental data as they arise.

**Conclusions:**

STI-GMaS represents the first software designed explicitly for *in-silico* simulation of STI models by non-theoreticians, thus presenting a novel route to bridging the gap between computational and clinical/experimental disciplines. With the propensity for model reuse and extension, there is much scope within STI-GMaS to allow clinical and experimental studies to inform model inputs and drive future model development. Many of the modelling paradigms and software design principles deployed to date transfer readily to other STIs, both bacterial and viral; forthcoming releases of STI-GMaS will extend the software to incorporate a more diverse range of infections.

## Background

In this article we describe the recent development of a novel software environment for modelling of sexually-transmitted infections (STIs). Since STI progression cannot be monitored within humans directly without being highly invasive and expensive, mathematical modelling is a valuable strategy in elucidating infection dynamics and evaluating the potential impact of new interventions [1]. Within-host modelling of STIs still being a relatively new field, determining new treatments currently relies primarily on animal trials. While some authors have sought to integrate mathematical models with data from animal experiments [2-5], there has to date been relatively little uptake of mathematical models by non-theoreticians, attributable (at least in part) to the fact that dynamic simulation of mathematical models relies upon the researcher having sufficient knowledge of mathematical techniques and computational strategies to generate simulations relevant to their specific studies. To enhance the relevance of model outputs, and strengthen the uptake of such models, it is invaluable to enable the investigator to run simulations of a range of models with easily customisable parameter sets and user-friendly visualisation of results.

We focus here upon the representative case study of *C. trachomatis* infection—the most common sexually-transmitted bacterial pathogen of humans, with over 105 million new adult cases occurring worldwide each year [6]. Due to its lengthy (6–14 day) incubation period and its high rate of asymptomatic infection, a large reservoir of *C. trachomatis* infection can accumulate in the lower genital tract of both men and women, without early detection. This reservoir presents a risk of an infection ascending to the upper genital tract with resultant adverse effects on reproductive health, including serious complications such as pelvic inflammatory disease (PID), tubal factor infertility and ectopic pregnancies. Although *C. trachomatis* affects men, women and infants, women bear the brunt of the chlamydial burden due to a higher risk of adverse reproductive consequences. We focus here upon *C. trachomatis* infection in females.

**Figure 1:**
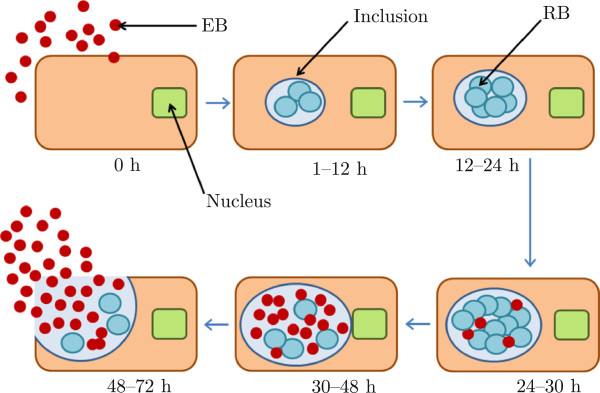
**The *C. trachomatis* replication cycle.** Initially a healthy epithelial cell is in the neighbourhood of extracellular chlamydiae. The cell becomes infected by a single EB, which adheres to the surface of the cell (illustrated at 0 hours). The EB is quickly engulfed by the cell and converts to the RB form within an inclusion (illustrated at 1–12 hours). RBs multiply approximately 500-fold by binary fission, and eventually convert back to the infectious EB form. The cell ultimately lyses (48–72 hours), releasing these infectious EBs.

*C. trachomatis* is an obligate, intracellular bacterium; *i.e.* its replication relies upon internalisation by a host cell of the genital epithelium (as shown in Figure 1). The bacterium exists in two forms: the infectious form, termed the *elementary body* (EB); and the intracellular replicative form, termed the *reticulate body* (RB). The EB is thought to be metabolically inert until it attaches to, and is endocytosed by, a susceptible host epithelial cell. After the EB attaches, it is rapidly ingested by an enhanced phagocytic process similar to ordinary bacterial phagocytosis [7]. Within two hours of internalisation, the EB differentiates into the metabolically active RB form. The RB then multiplies 200–500-fold by binary fission [8], before converting back into the infectious EB form. The host cell eventually lyses, releasing the new EBs back into the genital tract. The infection cycle then begins anew.

A common starting point for many recent within-host models of *C. trachomatis* is the model of [9], which uses ordinary differential equations (ODEs) to describe the temporal evolution of the chlamydial infection at the tissue-scale. The model incorporates infection of epithelial cells by EBs at a rate proportional to the concentration of the infection; birth, natural death and lysis of epithelial cells at prescribed rates; removal by macrophages of extracellular EBs; and removal of infected cells by elements of the cell-mediated immune system. This model provides a robust foundation for modelling of *C. trachomatis*, and has since been extended by many authors, including [10] who incorporated a more thorough multi-stage description of the intracellular EB replication process.

Recent studies by Mallet and co-workers [2,11-13] have placed greater focus upon understanding the processes through which *C. trachomatis* infection ascends the cervix. To incorporate the spatial dependence of extracellular quantities, the ODE model of [9] is replaced with a partial differential equation (PDE) analogue; extracellular particles are generally considered to move diffusively, and the models are able to incorporate *e.g.* chemotactic terms to capture migration of immune cells in response to diffusible chemical cues.

While PDE models succeed in qualitatively capturing the ascension of the infection, they lack a robust description of cell-scale phenomena. In the model of [2], the above PDE description of extracellular chlamydiae is combined with a cell-based description of the epithelium, in which each cell is modelled via a cellular automaton whose behaviour is governed by prescribed probabilistic rules. We further describe and extend this hybrid model in the forthcoming sections.

In the remainder of this article, we describe the development of a new software environment for easy simulation of a range of STI models, focusing on *C. trachomatis* in particular. We then illustrate the use of the software through a simple extension of the model of [2] to describe immunisation of adolescent females. We then describe scope for extensions of the software beyond the currently available models, and to describe other STIs.

## Implementation

To promote the use of mathematical and compuational models by clinical and experimental researchers, the authors have developed new software for easy STI model simulation. This software environment, named STI-GMaS (Sexually-Transmitted Infections – Graphical Modelling and Simulation) has been designed with accessibility and extensibility at its core, and makes progress toward thorough curation and annotation of models to clarify nomenclature, and maximise coherence between different models and ultimately across multiple infections.

STI-GMaS is built upon a framework of existing tools from within the Virtual Physiological Human (VPH) Toolkit—an online repository of tools for interdisciplinary modelling of the human physiome [14]. Integration with the Toolkit provides direct access to a number of standards for model development, annotation and validation, which (in the long-term) will allow STI-GMaS to be integrated with a range of other software for simulation of the wider physiome. Model simulations within STI-GMaS are implemented through new and existing finite-element tools provided by the open-source CHASTE (Cancer, Heart and Soft Tissue Environment) project [15-17], whose use provides on-going development in terms of diversity of tools, computational efficiency and cross-platform support. Furthermore, CHASTE’s delevopment deploys a test-oriented approach that ensures that algorithms are rigorously and regularly validated [16]; STI-GMaS benefits directly from this testing, and comparison with published results is performed for any newly added model, to ensure that simulation results are numerically accurate. For details of the implementation and testing of CHASTE itself, the reader is directed to [15-17].

**Figure 2:**
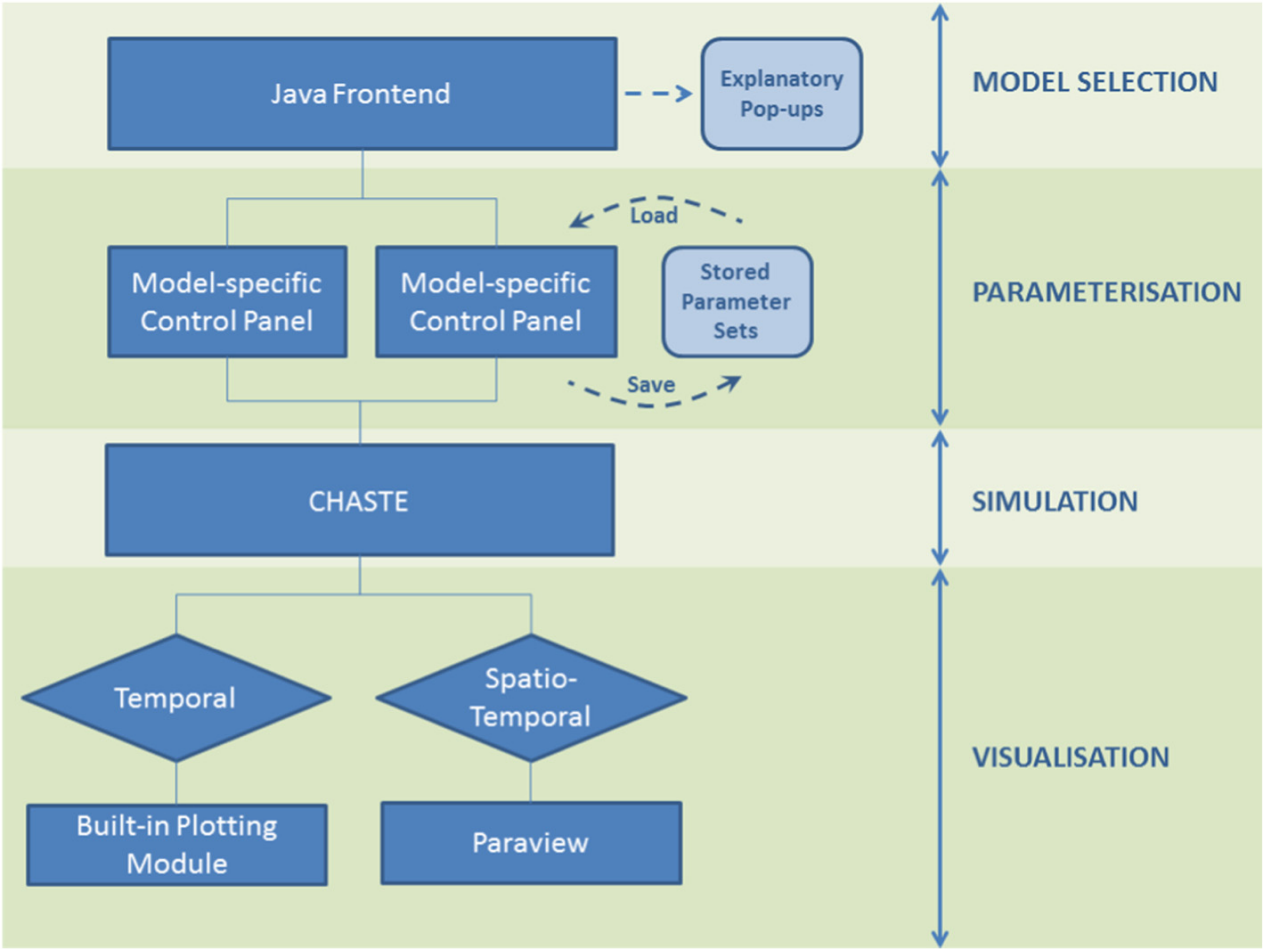
**The STI-GMaS workflow.** A user-friendly graphical user interface (written in Java) enables easy model selection and parameterisation, and allows simulation of underlying models to be initiated using CHASTE. Results are passed to one of two visualisation tools, selected dependent upon whether the model contains spatial information.

**Figure 3:**
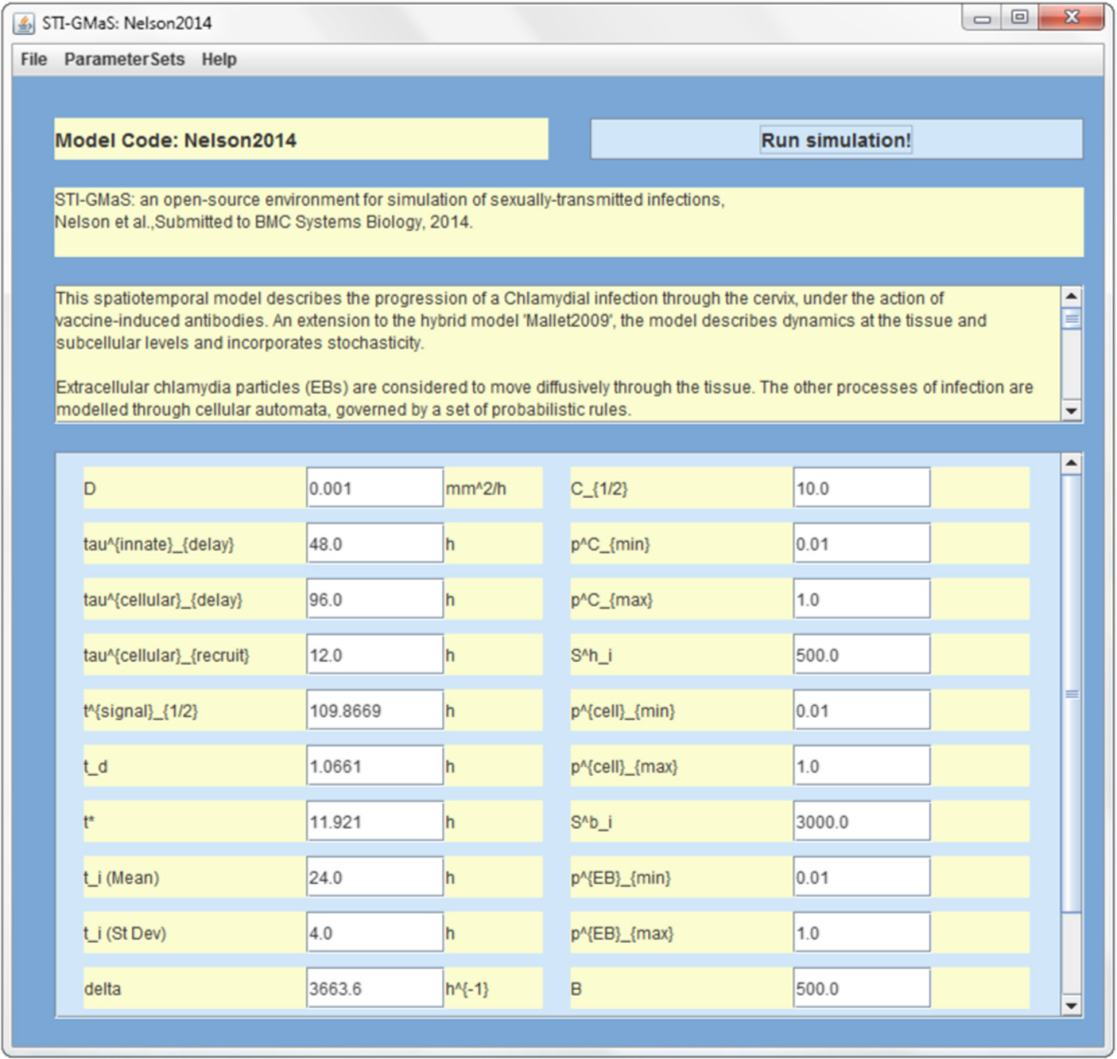
**A typical STI-GMaS model window.** Each incorporated model is accessed through a control panel, which provides a citation and brief description of the model, and allows for setting of each of the model’s parameters prior to running of a new simulation. Parameter nomenclature is consistent with the model’s original publication; hovering over any parameter will display the meaning of that parameter as hover help.

STI-GMaS integrates the outputs of these CHASTE simulations with visualisation tools to illustrate the biological phenomena that the models predict. These components are integrated via a graphical user interface (written in Java), which is also responsible for providing clear model annotation and parameterisation to enable models to be easily redeployed by the non-theoretical user. STI-GMaS is available on all 64-bit CHASTE-compatible operating systems, including linux, Mac OS X and Windows.Figure 2 illustrates the workflow via which the various components of STI-GMaS are integrated. On loading STI-GMaS, the user is presented with a list of currently available models, categorised as either temporal or spatio-temporal, and complemented with pop-up windows to inform non-theoretical users as to which class of models is most appropriate for their study. On selecting a model, the user arrives at a model-specific control panel (Figure 3) through which they can select from a number of default parameter sets (representing biologically important behaviours) or select new parameters to examine potentially new phenomena, allowing for a more thorough investigation of the model than may have been afforded by its original publication. All user-defined parameter sets can be saved and re-loaded for future simulations.

The implementation of STI-GMaS has considered two broad classes of end user: the clinician/experimentalist, who will be able to run new simulations of existing models easily; and the theoretician, for whom the source code is accessible for encoding of new models. To facilitate the latter, templates have been created within STI-GMaS to govern model parameteristion and enforce a minimum level of model annotation. New models will be required to meet these standards prior to being included in future releases; STI-GMaS developers will work alongside authors of new models to incorporate these as they arise.

STI-GMaS is distributed as an open-source ‘bolt-on’ of CHASTE, is licensed under CHASTE’s 3-clause BSD license, and can be downloaded via the STI-GMaS website, or directly from the CHASTE homepage [18].

## Results

Here, we demonstrate the use of STI-GMaS as an investigative tool for future interventions and treatment. We consider a scenario in which adolescent females are to be immunised with a *Chlamydia* vaccine prior to their sexual debut, to circumvent the development of inflammation in the Fallopian tubes: a common precursor to PID. The aims of the vaccination could be achieved by halting the ascension of the *C. trachomatis* organism through the genital tract, and by reducing the level of infection within the Fallopian tubes to a level below that required to trigger an inflammatory response.

The modelling assumptions in the design of the vaccination intervention are: (i) it takes at least 24 hours for white blood cells to enter the tissue at the site of infection, (ii) the vaccine elicits a strong cell-mediated immune response that becomes effective more quickly as a result of the expansion of the antigen-specific T cell population, and (iii) the vaccine enhances humoral immunity such that an antibody response is already present in the cervical secretions. The antibodies can combat the infection in two ways: either by neutralising extracellular chlamydiae, preventing attachment to host cells; or by stimulating phagocytosis of extracellular EBs, removing them from the system.

**Figure 4:**
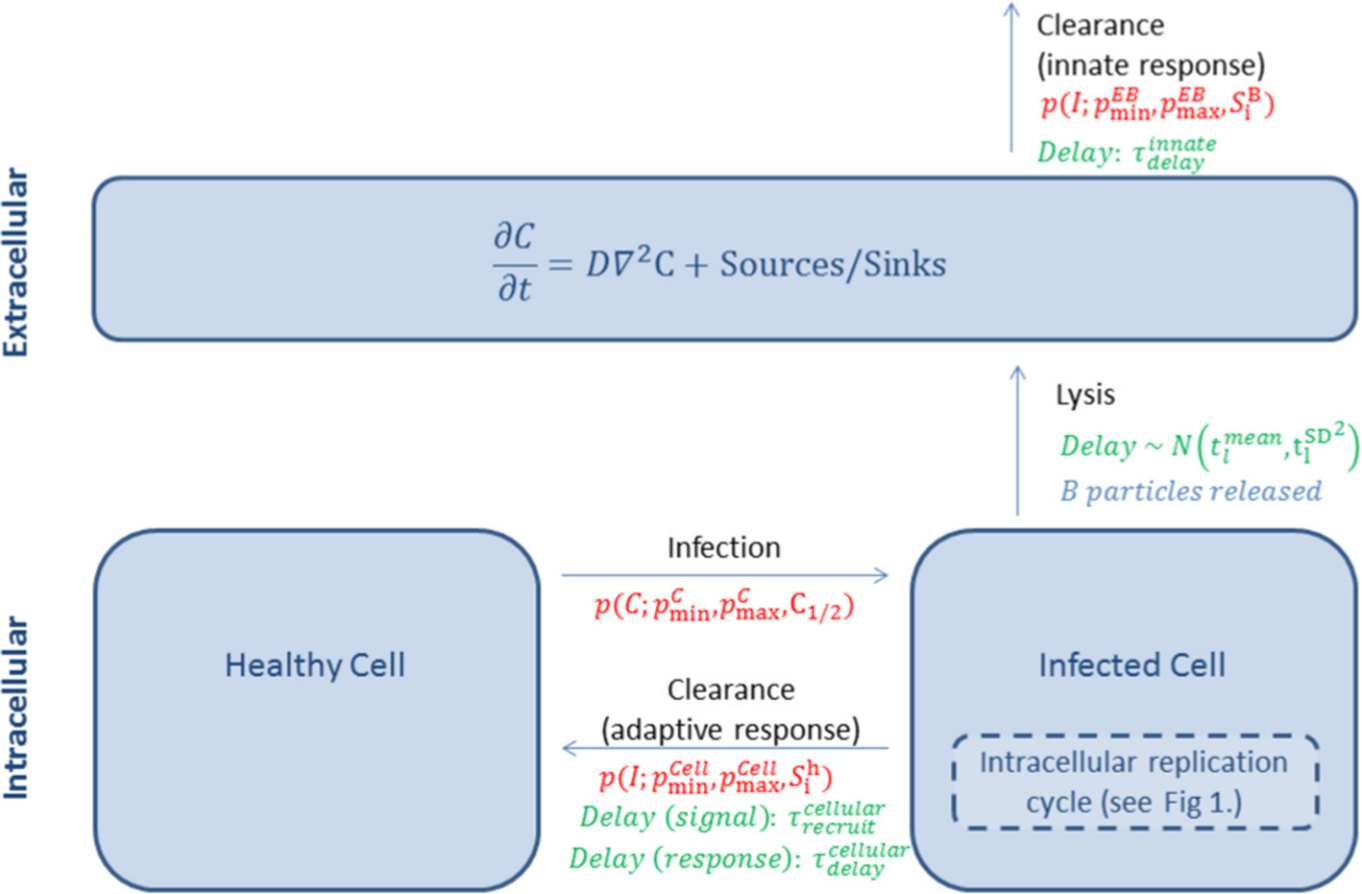
**Schema of the model of Mallet (2009).** Illustrated are the infection and clearance events incorporated in the model of [2], showing parameterisation in the nomenclature of the model’s original publication. Cell infection occurs as a function of local extracellular particle number (*C*), while clearance events are governed by a local infection signal (*I*), computed as a historic measure of the number of neighbouring cells to have been infected. Each event occurs with likelihood given by (1), for parameters and inputs shown in red; associated delays are given in green. For a full list of parameter values, see [2].

We model the intervention through an extension of the hybrid PDE–CA model of [2] (shown schematically in Figure 4). In the following, we deploy nomenclature consistent with [2]. In this model, extracellular EBs move diffusively with concentration *C*(**x**,*t*), and healthy cells become infected with probability proportional to the number of extracellular EBs in their vicinity (as discussed below). On infection, a cell is allocated a length of time until lysis, drawn from an appropriate normal distribution, since infection prevents natural death by apoptosis. On lysis, *B* new EBs are released, which appear as a source term in the extracellular PDE. After lysis, the void which remains in the cell monolayer is considered to be immediately filled by a new healthy cell. The model accounts for both innate and cell-mediated immune responses: as the density of infected cells increases, so does the strength of an emitted chemical infection signal (*I*), which is used to calculate the likelihood with which immune responses clear extracellular EBs or remove the infected cell. The signal strength at a given cell location increases while that cell remains infected; this infection signal is also transmitted to neighbouring cells across cell boundaries. If there are no infected cells in a given neighbourhood, the local infection signal is subject to exponential decay at a prescribed rate. The model incorporates delays between cell infection, activation of the response and recruitment of immune cells to the infected cell location. Each of the above infection and clearance events occurs probabilistically with likelihood given by

(1) $$\begin{array}{lll} \text{Pr}\left(\text{event}\right) & =p\left(X;p_{\min},p_{\max},X_{1/2}\right)\; & \;\\ & \equiv \frac{p_{\min}p_{\max}e^{\text{kX}}}{p_{\max}+p_{\min}\left(e^{\text{kX}}-1\right)},\; \end{array}$$

with

(2) $$k=\ln{\left(\frac{0.5\left(p_{\max}-p_{\min}\right)}{p_{\max}p_{\min}-0.5p_{\min}}\right)}^{1/X_{1/2}}.$$

In (1–2), *X* represents either the local extracellular EB concentration (*C*) or infection signal level (*I*), and *X*_1/2_ is the value of *X* for which Pr(*event*)=0.5. Each infection or clearance event is therefore described by three parameters: *p*_min_, *p*_max_ and *X*_1/2_. We direct the reader to [2] for further information regarding model parameters and their values.

We now introduce a new variable *A* to represent the vaccine-induced antibody level, and allow the model’s original parameters to vary with *A* in the manner described by (1). We assume that in the presence of high levels of antibody, the maximum probability of cell infection is reduced, and the minimum probability of EB clearance is increased. In the nomenclature of the original model, we let

(3) $$\begin{array}{ll} p_{\max}^{\text{cell}} & ∼1-p\left(A;p_{\min}^{A},p_{\max}^{A},A_{1/2}\right),\; \end{array}$$

(4) $$\begin{array}{ll} p_{\min}^{\text{EB}} & ∼p\left(A;p_{\min}^{A},p_{\max}^{A},A_{1/2}\right).\; \end{array}$$

The extent to which these quantities are adjusted is suitable for later fitting to experimental data through tuning of the new parameters $p_{\min}^{A}$, $p_{\max}^{A}$ and *A*_1/2_. We examine the progression of the infection under three antibody levels: no antibodies (*A*=0, *A*_1/2_=100; corresponding to no vaccination), moderate antibody levels (*A*=50, *A*_1/2_=100), and high antibody levels (*A*=100, *A*_1/2_=100). Typical values of other parameters are illustrated in Figure 3 (in which nomenclature is as given in the original paper), and parameter sets are provided in full within the STI-GMaS software as model ‘Nelson2014’.

**Figure 5:**
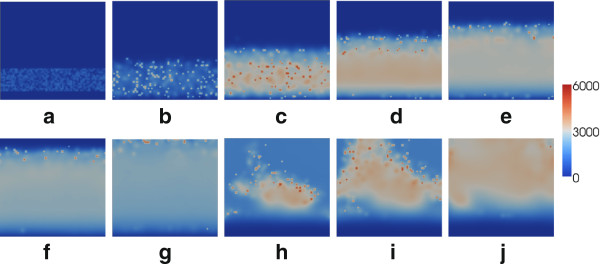
**Ascension of *C. trachomatis* in the cervix in the absence of vaccination.** Results illustrated are the number of extracellular chlamydiae at each spatial location at **(a)** 0 hours, **(b)** 24 hours, **(c)** 48 hours, **(d)** 72 hours, **(e)** 96 hours, **(f)** 120 hours, **(g)** 144 hours, **(h)** 372 hours, **(i)** 384 hours and **(j)** 408 hours. The infection quickly spreads through the cervix and beyond, with reinfections occurring periodically in the lower cervix due to lysis of infected epithelial cells.

**Figure 6:**
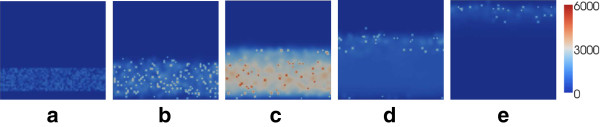
**Progression of *C. trachomatis* infection through the cervix under a moderate vaccine-induced antibody level.** Results illustrated are the number of extracellular chlamydiae at each spatial location at **(a)** 0 hours, **(b)** 24 hours, **(c)** 48 hours, **(d)** 72 hours and **(e)** 144 hours. While the majority of the extracellular chlamydiae are removed due to the antibody, some chlamydiae do ascend to above the cervix.

**Figure 7:**
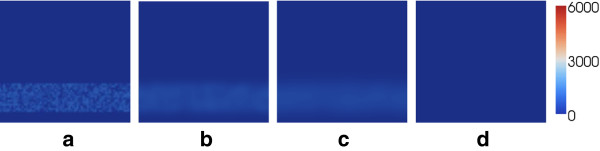
**Evolution of *C. trachomatis* infection in the cervix under a high vaccine-induced antibody level.** Results illustrated are the number of extracellular chlamydiae at each spatial location at **(a)** 0 hours, **(b)** 20 hours, **(c)** 40 hours and **(d)** 60 hours. The infection is quickly removed by the immune system, and does not ascend to above the cervix.

Figures 5, 6 and 7 illustrate the ascension of EBs in each of these scenarios. The domain illustrated represents the cervical epithelium, oriented with the vaginal boundary at the base. The model is subject to periodic boundary conditions on the lateral boundaries, to recover the 3D geometry. Following [2], we impose *C*=0 on the lower boundary, since the lower genital tract is inhospitable to chlamydial infection, and impose a zero-flux condition on the top boundary to represent the effects of the cervical mucus plug. In each simulation, a deposit of extracellular chlamydiae is initially randomly distributed in a stripe close to the lower boundary.

In the absence of the antibody (Figure 5), the infection builds rapidly and ascends the cervix in a manner reminiscent of a travelling wave. The infection progresses to above the cervix (not modelled here), with potentially serious implications upon reproductive health. Infection in the cervix itself is also maintained, with lysis of infected cells generating recurring peaks in EB number (Figure 5(h)). With a moderate antibody level (Figure 6), the short-term behaviour is similar to that of Figure 5; however, after 72 hours the majority of the EBs have been successfully removed as a result of the antibody stimulating the immune response and hindering cell infection. While the situation is somewhat improved by the presence of the antibody, a small number of EBs do still ascend to the upper cervical boundary (Figure 6(e)). For a high antibody level (Figure 7) the infectious extracellular chlamydiae are quickly removed by the immune response; the infection is successfully prevented from ascending to the upper reproductive tract. For the demonstrative parameters used here, the infection is cleared within 60 hours; a timescale similar to those observed in related animal inoculation experiments [19].

## Discussion

The current release of STI-GMaS (version 1.2) incorporates three *Chlamydia* models: that of [9], a temporal model operating on the tissue scale, the outputs of which compare readily to the results of many animal experiments; that of [2], which elucidates the spatial ascension of an infection up the female genital tract; and a new extension of the latter model (described above) to examine the effectiveness of a hypothetical vaccine. This vaccine model demonstrates how existing models can be readily adapted and reused through STI-GMaS, and exhibits much scope for coupling to laboratory experiments to inform parameter values. With parameter values more accurately determined to model specific vaccines, the model will be in a position to address questions regarding appropriate vaccine dosage and optimisation of immune response times to remove infections efficiently.

While this article has focused upon the use of STI-GMaS for *in silico* experimentation, there is also great scope for its use as part of *e.g.* Approximate Bayesian Computation (ABC) schemes for inferring model parameters from data [20-22]. These schemes weight parameters according to the closeness of a forward simulation of a model (such as those produced by STI-GMaS) to true data, allowing for good parameter estimation even in the presence of extremely complicated disease dynamics. Hence, STI-GMaS has the potential to facilitate model simulation, parameterisation and inference within the same software package.

The design of STI-GMaS has extensibility at its core: further models will be added to the software in due course, subject to strict standards of clear model annotation and parameterisation being demonstrably met. The framework for incorporating these models is now well-defined, with templates in use for creation of codes for CHASTE simulations and for the graphical user interface itself. While we do not expect non-theoretical users to add new models to the software themselves, these templates ease the developers’ task of introducing new models as they arise, and the environment’s open source design makes the corresponding codes accessible to researchers of any background who do wish to become more familiar with the computational principles.

The models discussed herein exhibit great scope for future extension. Currently, a key limitation of spatial models lies in their focus upon only cervical tissue. Expansion of the modelled domain to include regions up to the Fallopian tubes, would allow for investigations of scarring, inflammation and PID. One direct target for such a model is the “arrested immunity” hypothesis proposed by [23], which suggests that although early antibiotic treatment can cure the chlamydial infection, it can also interrupt the development of immunity. Consequently, the prevalence of *Chlamydia* in the population does not decline but actually increases. However, early treatment can prevent the development of PID. The experimentalist may therefore question whether there exists a time frame during which antibiotic intervention can prevent PID but also allow the host to develop immunity, potentially leading to new antibiotic interventions. The mathematical model presented here exhibits many of the requirements of such a theoretical investigation, although it does not currently represent adequate regions of the host anatomy.

We note that while work to date has focused upon within-host models of *Chlamydia*, targets for future development within the STI-GMaS environment are to incorporate both population-scale models, and models of other STIs. Many of the modelling techniques deployed here transfer directly to within-host models of other infections: models of gonorrhoea show particular similarity to those described above and, while having a different pathogenesis to that of Chlamydia, syphilis and viral infections such as Herpes simplex virus, Human papillomavirus and viral hepatitis could be represented with models employing a highly similar process. Future work will examine the above infections in isolation, as well as co-infections, and will incoporate such models into the STI-GMaS software.

## Conclusions

The STI-GMaS environment represents, to the authors’ knowledge, the first software designed explicitly for *in silico* simulation of STI models by non-theoreticians. Albeit a growing field, mathematical modelling of STIs is in its relative infancy, with little uptake of models in biological domains. This software presents a novel route to bridging the gap between computational and clinical/experimental disciplines, and in doing so will stimulate dissemination and uptake of both existing and new theoretical models.

As described above, the implementation of Chlamydia models to date constitutes a template that can be easily extended to other STIs. Such extensions will be included in future releases. A key target for future development will be to include within STI-GMaS a full geometric desciption of the genital tract, which can be deployed readily within new models, regardless of the infection under consideration. In parallel, key parameters will be identified in each of the key subtissues and made available across models. In doing so, STI-GMaS will improve upon the current necessity for models to be heavily relient on phenomenological parameter values.

We remark that, while STI-GMaS has made headway in making computational models more accessible, a true bridging of disciplines would benefit greatly from a bidirectional exchange of information. The task of making experimental data available through repositories with robust annotation is considerable and unresolved, and has been the focus of many other VPH-related initiatives; see *e.g.*[24] for details. Such integration of experimental data remains a long-term target for STI-GMaS.

## Availability and requirements

**Project name**: STI-GMaS

**Project home page**: http://www.sti-gmas.org

**Operating systems**: Linux, Mac OS X, Windows (64 bit)

**Programming language**: C++, Java

**Other requirements**: Paraview

**License**: 3-clause BSD

**Restrictions to use by non-academics**: none.

## Competing interests

The authors declare that they have no competing interests.

## Authors’ contributions

MRN and KJS led implementation of the software; BSB, DGM, DPS and RGR guided development of contributory models. All authors contributed equally to production of this article. All authors read and approved the final manuscript.
